# Safety and efficacy of carbon nanoparticle suspension injection and indocyanine green tracer-guided lymph node dissection during robotic distal gastrectomy in patients with gastric cancer

**DOI:** 10.1007/s00464-021-08630-8

**Published:** 2021-07-12

**Authors:** Yuan Tian, Yecheng Lin, Honghai Guo, Yiyang Hu, Yong Li, Liqiao Fan, Xuefeng Zhao, Dong Wang, Bibo Tan, Qun Zhao

**Affiliations:** Third Surgery Department, The Fourth Hospital of Hebei Medical University, Shijiazhuang, Hebei China

**Keywords:** Carbon nanosuspension, Indocyanine green, Lymph node dissection, Robotic distal gastrectomy

## Abstract

**Background:**

There is a lack of comparative analyses on the use of carbon nanoparticle suspension injection (CNSI) and indocyanine green (ICG) tracer technology for lymph node detection and their perioperative safety in robotic radical gastrectomy.

**Methods:**

A retrospective analysis was performed on patients who underwent robotic distal gastrectomy between November 2019 and November 2020. Patients were assigned to the CNSI group, the ICG group, or the control group. The number of lymph nodes detected, number of lymph nodes detected at each station, number of micro lymph nodes detected, rate of lymph node metastasis, and inoperative and postoperative recovery were compared.

**Results:**

Of the 93 patients analyzed, 34 were in the CNSI group, 27 were in the ICG group, and 32 were in the control group. The mean number of lymph nodes retrieved in the CNSI group (48.44) was higher than that in the ICG (39.19) and control (35.28) groups (*P* = 0.004; *P* < 0.001), and there was no difference between the ICG and control groups (*P* = 0.102). The mean number of micro lymph nodes retrieved in the CNSI group (13.24) was higher than that in the ICG (5.74) and control (5.66) groups (*P* < 0.001). The lymph node metastasis rates in the CNSI, ICG, and control groups were 5.03, 4.63, and 5.93%, respectively (*P* > 0.05).

**Conclusion:**

The effect of CNSI on lymph node dissection and sorting was better than that of ICG, and CNSI improved the surgical quality and reduced lymph node staging deviation to a greater extent. CNSI was better than ICG in terms of improving the number of micro lymph nodes detected.

D2 lymph node dissection has been shown to improve survival in patients with gastric cancer [1]. Good quality control is necessary for intraoperative D2 lymph node dissection, and the classification of lymph nodes in postoperative specimens is an important factor for the accurate pathological staging of gastric cancer [2]. Application of the lymph node tracer technique is an important way to improve the quality control of intraoperative lymph node dissection and correct postoperative lymph node staging deviation. As lymph node tracer, carbon nanoparticle suspension injection (CNSI) and indocyanine green (ICG) can be applied intraoperatively and can be used to sort lymph nodes in postoperative specimens [3–5]. Among them, ICG, as a special fluorescent agent, can be excited by light with a wavelength ranging from 750 to 810 nm and can emit near-infrared light with a wavelength of approximately 840 nm [6]. When injected outside the blood vessels, ICG binds to proteins and is found in the lymph, where it typically reaches the nearest draining lymph node within 15 min. After 1–2 h, ICG binds to regional lymph nodes and is deposited into macrophages. ICG fluorescence imaging technology is based on the above principles and requires special imaging equipment to trace the drainage of lymphatic vessels and lymph nodes. In addition, due to the different uptake rates of ICG in different tissues, this dye can effectively distinguish lymphatic tissue from gastric peripheral blood vessels, fat, pancreas, and other tissues during surgery. [7]. Moreover, Chen QY [8] and Kwon IG [9] have demonstrated the effectiveness and reliability of ICG injection given to patients under gastroscopy 1 day before surgery. CNSI is different from tattoo ink in that it consists of carbon nanoparticles with an average diameter of 150 nm, which ensures that these particles pass through the lymphatic vessels rather than blood capillaries due to their molecular size and permeability. Upon injection into the tissues around the tumor, carbon nanoparticles are rapidly engulfed by macrophages. The particles then enter the lymphatic vessels and accumulate in the lymph nodes, thus staining them black [10]. In addition, CNSI can be observed in vivo after approximately 3–4 months [11]. In recent years, both CNSI and ICG have gained acceptance among surgeons for minimally invasive procedures, such as laparoscopy and robotic surgery [12–14], but they have different effects when used as intraoperative lymph node/micro lymph node tracers and for postoperative lymph node sorting.

The use of robotic systems for surgical procedures has increased rapidly over the past 2 decades. The robot's flexible arm facilitated lymph node dissection [9]. However, an evaluation of the two tracers used in robotic distal gastrectomy has not been performed. This study therefore aimed to compare the efficacy of these tracers in lymph node harvesting and their perioperative safety during robotic ICG-guided and CNSI-guided radical gastrectomy procedures in patients with gastric cancer. It also aimed to identify the best lymph node tracer technology for robotic distal gastrectomy in patients with gastric cancer.

## Materials and methods

### Study design and participants

Data were collected from patients who underwent robotic distal gastrectomy at the Third Surgery Department of the Fourth Hospital of Hebei Medical University between November 2019 and November 2020. Patients in the CNSI group received an endoscopic injection of CNSI 1 day before surgery, patients in the ICG group received an endoscopic injection of ICG 1 day before surgery, and patients in the control group underwent surgery directly without any labeling. The following general clinical data of the patients in the three groups were analyzed: sex, age, body mass index (BMI), tumor diameter, clinical and pathological stages, Lauren classification, tumor location, and tumor differentiation degree.

The inclusion criteria were as follows: (1) received robotic distal gastrectomy; (2) gastric cancer was confirmed by postoperative pathology; (3) intraoperative and postoperative lymph node specimens were classified by the same group of surgeons; and (4) patients received an endoscopic injection of CNSI or ICG 1 day before surgery. The exclusion criteria were as follows: (1) distant metastases to the liver, lung, peritoneum, etc., found during the preoperative examination or intraoperative exploration; (2) required palliative surgical resection; (3) complications with tumors in other parts; (4) received neoadjuvant therapy (chemotherapy, targeted therapy, etc.) before surgery; and (5) emergency surgery for bleeding, perforation, obstruction, etc. This study complied with the requirements of the Declaration of Helsinki, and informed consent was obtained from patients who underwent relevant data analysis.

### Experimental drugs and methods

CNSI group: CNSI (50 mg/dose) was produced by *Chongqing Lesmei Pharmaceutical Co., Ltd.:* Carbon nanoparticles were marked in the endoscopy division 1 day before surgery, and CNSI was injected submucosally at 4 points (proximal side, distal side, and left and right sides) 0.5–1 cm from the tumor edge under endoscopy. The test dose for each point was approximately 0.25 ml.

ICG group: ICG (25 mg/dose) was produced by *Dandong Yichuang Pharmaceutical.* ICG was marked in the endoscopy division 1 day before surgery and injected submucosally at 4 points (proximal side, distal side, and left and right sides) 0.5–1 cm from the tumor edge under endoscopy. The test dose for each point was approximately 0.5 ml. Both procedures were performed by a designated medical practitioner.

### Surgical quality control

**Fig. 1:**
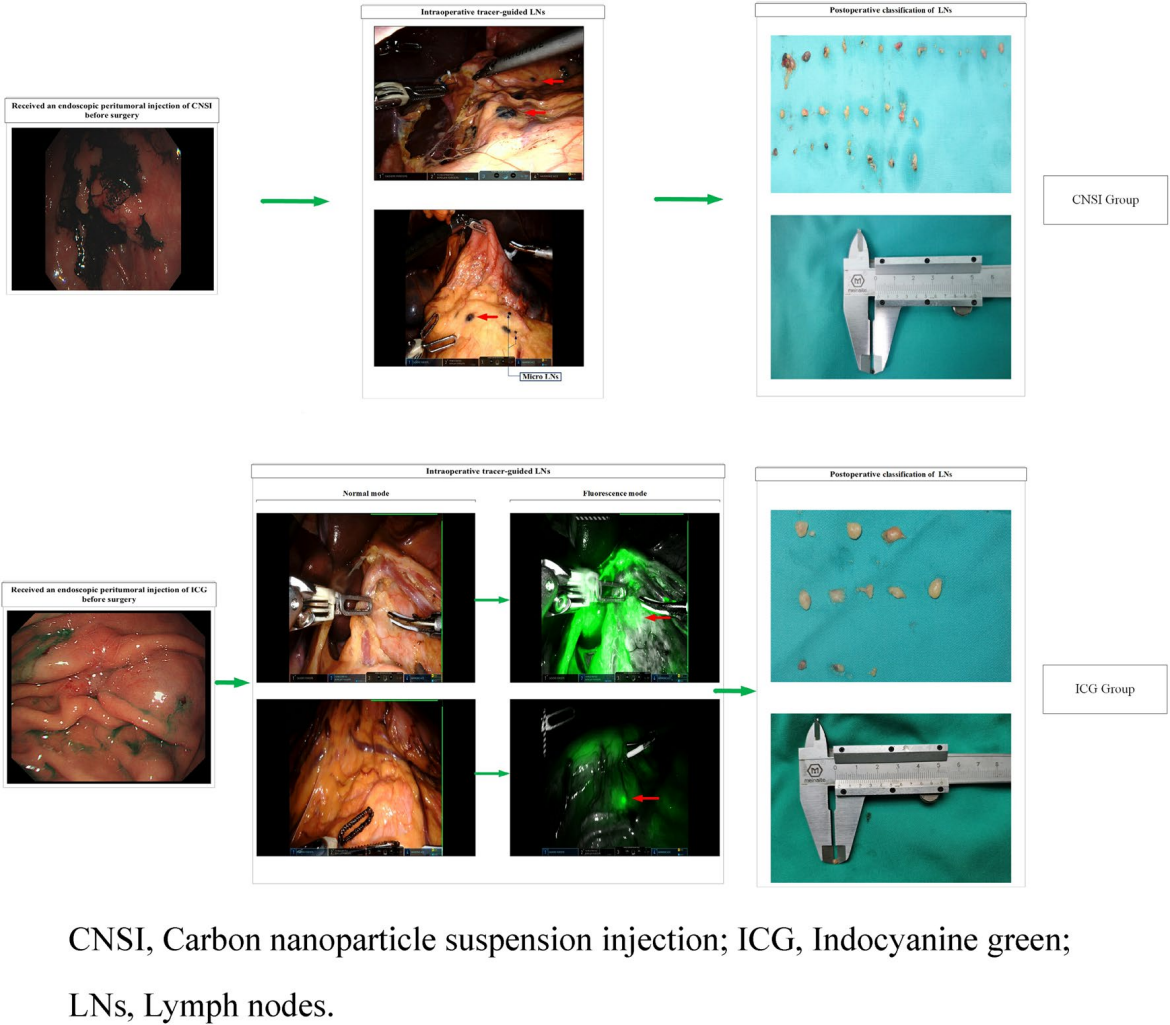
Procedures performed in the CNSI and ICG groups

All patients received D2 radical robotic distal subtotal gastrectomy, and the retrieval of lymph nodes intraoperatively was performed according to the Expert Consensus of Robotic Gastric Cancer Surgery (2015 edition) [15]. In the ICG group, the operator changed the fluorescence mode according to the specific conditions during the operation, and distal subtotal gastrectomy and D2 lymph node dissection were performed at the same time. Lymph node sorting was performed under direct visualization by designated experienced physicians (Fig. 1).

### Statistical analysis

Statistical analysis was performed using SPSS 20.0 software (SPSS Inc., IBM, Armonk, NY, USA). Measurement data are expressed as the mean and standard deviation (X ± S). The *F* test, *T* test, and analysis of variance (ANOVA) were used for comparisons. Counting data from each group were tested by the chi-square test, and the Fisher's exact probability test was used when necessary. Pairwise comparisons were performed with the least significant difference (LSD)-*t* test. A *P* value < 0.05 was considered statistically significant.

## Results

Ninety-three patients were included in the statistical analysis and were divided into the CNSI group (34 patients), ICG group (27 patients), and control group (32 patients). No significant differences were observed in sex, age, BMI, tumor diameter, clinical and pathological stage, Lauren classification or tumor location among the three groups (*P* > 0.05), which indicates that the baseline conditions of the three groups were comparable (Table 1). The CNSI group underwent CNSI tracer-guided robotic gastrectomy, the ICG group underwent ICG tracer-guided robotic gastrectomy, and the control group underwent conventional robotic gastrectomy.

**Table 1:** Basic characteristics of the three patient groups

	CNSI (*n* = 34)	ICG (*n* = 27)	Control (*n* = 32)	*F/*χ^2^	*P*
Sex					
Male	22	14	14	2.968	0.227
Female	12	13	18		
Age, years	56.91 ± 8.59	57.67 ± 10.48	58.59 ± 12.14	0.213	0.809
BMI	24.21 ± 2.59	23.76 ± 2.91	23.52 ± 2.85	0.525	0.593
Tumor diameter, cm	2.47 ± 1.42	2.93 ± 0.88	2.84 ± 1.28	1.211	0.303
Clinical stage					
I	11	12	12	1.056	0.901
II	15	9	13		
III	8	6	7		
Lauren classification					
Intestinal	7	11	8	6.685	0.153
Diffuse	18	9	10		
Mixed	9	7	14		
Differentiation degree					
Low	10	15	16	8.829	0.066
Medium	15	7	5		
Low-medium	9	5	11		
Tumor location					
Body	5	6	7	1.702	0.790
Angle	16	12	11		
Antrum	13	9	14		
Pathological stage					
I	8	13	10	4.814	0.307
II	16	7	14		
III	10	7	8		

*CNSI* carbon nanoparticle suspension injection, *ICG* indocyanine green, *BMI* body mass index

### Lymph node dissection

The mean number of lymph nodes retrieved in the CNSI group (48.44 ± 13.87) was higher than that in the ICG (39.19 ± 8.97) and control (35.28 ± 9.00) groups (*F* = 12.387, *P* < 0.001) (*T* = 3.002, *P* = 0.004/*T* = 4.542, *P* < 0.001), but the ICG group was not different than the control group in this regard (*T* = 1.663, *P* = 0.102).

The mean number of retrieved micro lymph nodes in the CNSI group was 13.24 ± 4.45, which was significantly higher than that in the ICG (5.74 ± 3.11) and control (5.66 ± 3.28) groups (*F* = 12.387, *P* < 0.001) (*T* = 3.002, *P* = 0.004/*T* = 4.542, *P* < 0.001), but no difference in retrieved micro lymph nodes was observed between the ICG and control groups (*T* = 1.663, *P* = 0.102) (Table 2).

**Table 2:** Lymph node dissection in the three patient groups

	CNSI (*n* = 34)	ICG (*n* = 27)	Control (*n* = 32)	*F/*χ^2^	*P*
Mean number of retrieved LNs	48.44 ± 13.87	39.19 ± 8.97	35.28 ± 9.00	12.387	< 0.001
Mean number of retrieved micro LNs	13.24 ± 4.45	5.74 ± 3.11	5.66 ± 3.28	44.564	< 0.001
LN metastasis rate	5.03% (83/1647)	4.63% (49/1058)	5.93% (67/1129)	2.018	0.365

*CNSI* carbon nanoparticle suspension injection, *ICG* indocyanine green, *LN(s)* lymph node(s)

### Lymph node metastasis

A comparison of the rate of lymph node metastasis among the three groups indicated that the rate of lymph node metastasis in the CNSI [5.03% (83/1647)] and ICG [4.63% (49/1058)] groups was not significantly higher than that in the control group [5.93% (67/1129)] regardless of the resection method used (*P* > 0.05) (χ2 = 0.231, *P* = 0.631) (χ2 = 1.050, *P* = 0.306) (χ2 = 0.1.846, *P* = 0.174). Moreover, the rate of stained lymph node metastasis in the CNSI group was 5.70% (59/1035), which was not significantly different from that of unstained lymph nodes [3.92% (24/612)] (χ2 = 2.543, *P* = 0.111) (Table 2).

### Number of detected lymph nodes at each station

The numbers of lymph nodes detected at the same lymph node station in the CNSI and ICG groups were not higher than those in the control group, as shown at stations 1, 4sb, and 7 (*P* > 0.05). However, the numbers of lymph nodes dissected at the same lymph node station, especially at stations 3 ([12.21 ± 5.32] vs. [7.38 ± 2.43]; *P* < 0.001), 5 ([1.97 ± 0.87] vs. [1.56 ± 0.67]; *P* = 0.037), 6 ([5.47 ± 2.50] vs. [2.81 ± 1.80]; *P* < 0.001), 8a ([1.97 ± 0.90] vs. [1.44 ± 0.62]; *P* = 0.007), 9 ([1.85 ± 0.89] vs. [1.41 ± 0.61]; *P* = 0.022), and 12a ([1.80 ± 1.04] vs. [1.19 ± 0.64]; *P* = 0.006), were higher in the CNSI group than in the control group.

**Fig. 2:**
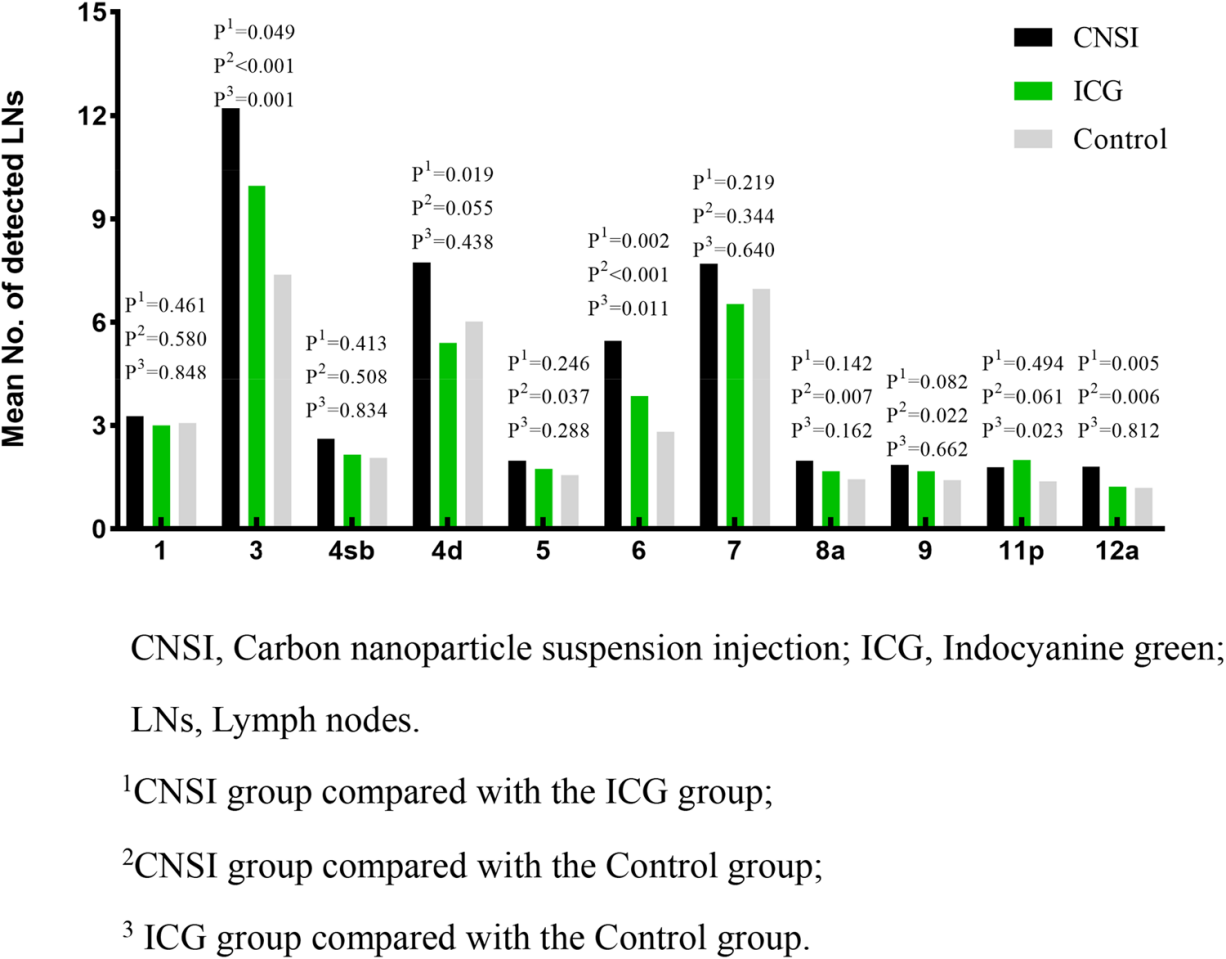
No. of detected LNs at each station

The numbers of lymph nodes dissected at stations 3 ([9.96 ± 3.31] vs. [7.38 ± 2.43]; *P* = 0.001), 6 ([3.85 ± 1.20] vs. [2.81 ± 1.80]; *P* = 0.011), and 11p ([2.00 ± 1.30] vs. [1.38 ± 0.71]; *P* = 0.023) were higher in the ICG group than in the control group. In addition, the numbers of lymph nodes dissected at stations 3 ([12.21 ± 5.32] vs. [9.96 ± 3.31]; *P* = 0.021), 4d ([7.74 ± 4.11] vs. [5.41 ± 3.45]; *P* = 0.019), 6 ([5.47 ± 2.50] vs. [3.85 ± 1.20]; *P* = 0.049), and 12a ([1.80 ± 1.04] vs. [1.22 ± 0.42]; *P* = 0.005) were higher in the CNSI group than in the ICG group (Fig. 2).

### Intraoperative situation

The mean operative time of the CNSI group was not significantly different from that of the ICG and control groups [(228.06 ± 30.89) min vs. (230.52 ± 20.71) min vs. (238.78 ± 34.44) min; *P* = 0.319]. The mean blood volume loss during surgery was not significantly different among the three groups [(37.50 ± 15.14) ml vs. (40.19 ± 18.21) ml vs. (44.69 ± 13.73) ml; *P* = 0.177] (Table 3).

**Table 3:** Intraoperative conditions of the three patient groups

	CNSI (*n* = 34)	ICG (*n* = 27)	Control (*n* = 32)	*F*	*P*
Mean operative time, min	228.06 ± 30.89	230.52 ± 20.71	238.78 ± 34.44	1.156	0.319
Mean blood loss during surgery, ml	37.50 ± 15.14	40.19 ± 18.21	44.69 ± 13.73	1.764	0.177

*CNSI* carbon nanoparticle suspension injection, *ICG* indocyanine green

### Postoperative situation

No significant difference was found among the three groups of patients in terms of exhaust time after surgery [CNSI group (1.68 ± 0.73) d vs. ICG group (1.89 ± 0.70) d vs. control group (1.84 ± 0.72) d; *P* = 0.466], feeding time after surgery [CNSI group (4.00 ± 2.28) d vs. *t* ICG group (4.96 ± 3.57) d vs. control group (5.16 ± 3.80) d; *P* = 0.309], or duration of postoperative hospital stay [CNSI group (7.85 ± 3.47) d vs. ICG group (8.30 ± 3.64) d vs. control group (8.22 ± 3.63) d; *P* = 0.870]. In the CNSI group, 2 cases experienced postoperative gastrointestinal bleeding and 1 case experienced gastroparesis, while in the ICG group, 1 case had postoperative gastrointestinal bleeding and 1 case had anastomotic fistula. In the control group, 1 case had postoperative abdominal infection and 2 cases had anastomotic fistula. All the complications in the patients described above resolved after conservative treatment, and no significant differences were found among the CNSI, ICG, and control groups in the total incidence of postoperative complications [3 of 34 patients (8.82%) vs. 2 of 27 patients (7.41%) vs. 3 of 32 patients (9.38%); *P* = 0.936]. (Table 4).

**Table 4:** Postoperative recovery in the three patient groups

	CNSI (*n* = 34)	ICG (*n* = 27)	Control (*n* = 32)	*F/*χ2	*P*
Mean exhaust time after surgery, d	1.68 ± 0.73	1.89 ± 0.70	1.84 ± 0.72	0.769	0.466
Mean feeding time after surgery, d	4.00 ± 2.28	4.96 ± 3.57	5.16 ± 3.80	1.189	0.309
Mean duration of postoperative hospital stay, d	7.85 ± 3.47	8.30 ± 3.64	8.22 ± 3.63	0.139	0.870
Postoperative complication rate	8.82% (3/34)	7.41% (2/27)	9.38% (3/32)	0.132	0.936

*CNSI* carbon nanoparticle suspension injection, *ICG* indocyanine green

## Discussion

Studies have shown that the detection of more than 22 lymph nodes can have an important impact on the survival outcome of patients [16]. Therefore, to improve the survival rate of patients with gastric cancer, thorough intraoperative lymph node dissection and more standardized lymph node detection of postoperative specimens are necessary. In addition, Pan et al*.* [17] and Cianchi et al. [18] demonstrated the advantages of robotic surgery in lymph node dissection during radical gastrectomy. CNSI and ICG have been consistently accepted and recognized as new lymph node tracer techniques [9, 19]. In this study and in previous studies, CNSI and ICG were injected submucosally one day before surgery to produce a stabilizing effect [20, 21].

Lymph node staging has always been one of the most controversial components of gastric cancer staging. According to the staging system established by the American joint committee on cancer (AJCC) and the international union against cancer (UICC)/general rules for gastric cancer study (GRGCS), the pathological stage of lymph nodes in postoperative pathology is determined according to the number of lymph node metastases [22, 23]. Regardless of whether lymph node metastasis is present, thorough gastric lymph node resection is important for accurate staging, even subsequent treatment selection, and improved prognosis [24]. In this study, the mean number of lymph nodes retrieved in the CNSI group was higher than that in the ICG and control groups. After the injection of CNSI, the number of lymph nodes retrieved was significantly increased. Both tracers could be used to guide intraoperative lymph node detection and effectively avoid lymph node omission. Thus, the total number of lymph nodes retrieved is closer to an objective and real state to avoid deviation in lymph node staging and then more accurately guide treatment in the future.

However, although the ICG group was superior to the control group in the number of lymph nodes retrieved, there was no statistically significant difference. One study [8] revealed the advantage of ICG in increasing the number of lymph nodes retrieved. The reason that this was not observed in our study may be that in our study, fluorescence laparoscopic postoperative lymph node dissection was not applied to in vitro samples, so fluorescence laparoscopy for lymph node classification is the direction of subsequent research. Nevertheless, this method still has some disadvantages: one is that the procedure is very cumbersome, increasing the burden on operating room personnel, and the prolonged use of fluorescent laparoscopy increases the wear and tear of the instrument. The second is that in hospitals without fluorescent laparoscopy, it is almost impossible to use a robotic system to perform lymph node sorting in fluorescence imaging mode, and the advantage of ICG will be greatly reduced. In contrast, CNSI can be performed under direct vision to obtain better lymph node classification results.

The retrieval of micro lymph nodes has always been one of the difficulties in the lymph node classification of gastric cancer [25]. In a standard D2 lymph node dissection, if only normal or enlarged lymph nodes are detected, small metastatic lymph nodes may be missed, even if the number of lymph nodes dissected meets the standard. In previous studies, statistical analysis was performed on the diameter of lymph nodes around the stomach during radical gastrectomy, and those with a diameter less than 2 mm were regarded as micro lymph nodes. That study also found that CNSI could improve the detection of micro lymph nodes [26]. We also found that the number of micro lymph nodes detected in the CNSI group was obviously higher than that in the ICG and control groups, but the number in the ICG group was not higher than that in the control group. This result indicated that ICG did not have a beneficial effect on the detection of micro lymph nodes, while CNSI had a greater advantage in the detection of micro lymph nodes. Although it remains to be confirmed whether micro lymph nodes represent more lymph node metastasis, the detection of more micro lymph nodes will also increase the total number of lymph nodes retrieved, which means that the N stage will be more accurate, effectively avoiding staging deviations. However, no significant difference was observed in the number of metastatic lymph nodes among the three groups, and no significant difference was found in the metastasis rate of stained lymph nodes compared with unstained lymph nodes in the CNSI group. This indicates that neither CNSI nor ICG was helpful for tracing metastatic lymph nodes.

Bao et al. [27] reported that lymphatic drainage of the stomach is much more complex than that of ectodermal organs. Gastric lymphatic flow is multidirectional, which results in multiple lymph nodes with skip metastasis. Most positive nodes occur in the N1 compartment, with frequencies of 79.6–85.7% based on the tumor site. Among skip metastases, stations 7, 8a, 9, and 11p were the most common sites. We also analyzed the numbers of detected lymph nodes at each station. CNSI and ICG could increase the dissection rate of lymph nodes in the N1 compartment, and CNSI could increase the dissection rate of lymph nodes at stations 3, 4, 5, and 6. ICG could increase the dissection rate of lymph nodes at stations 3 and 6, but the number of lymph nodes retrieved after CNSI was higher than that retrieved after ICG, and the effect was more significant. CNSI could also increase the number of retrieved lymph nodes at stations 8a, 9, and 12a in the N2 compartment, and ICG could increase the number of retrieved lymph nodes at station 11p; however, neither CNSI nor ICG has obvious advantages for retrieved nodes at stations 1, 4sb, and 7. Nonetheless, it is not difficult to conclude that the overall effect of CNSI on the retrieval of lymph nodes at each station is better than that of ICG. In addition, regardless of whether CNSI or ICG was used, no stained lymph nodes were found, which may be due to the randomness of gastric cancer lymph node metastases or the presence of lymphatic vessel tumor emboli in the block [27]. The tracer cannot drain at this point, and therefore, although both can be used to guide lymph node dissection, neither CNSI nor ICG can be relied on alone.

Trace extravasation was also found during the operation. After the injection of ICG, the operator could still choose either fluorescence mode or normal mode independently to avoid the effect of the agent on the operative field. However, CNSI can remain in the body for long periods of time, and thus, extravasation will affect the operative field to some extent.

However, no significant difference was seen among the three groups of patients in terms of the operative time, bleeding during surgery, exhaust time after surgery, feeding time after surgery, duration of postoperative hospital stay, or total incidence of postoperative complications, which was consistent with relevant studies by Yan et al. [10] and Shoji et al. [28]. Moreover, the endoscopic injection of CNSI or ICG one day before surgery did not increase the risk of intraoperative bleeding or affect postoperative recovery. Of course, we need long-term follow-up results to evaluate the long-term efficacy and whether these agents will affect the long-term prognosis of patients.

In summary, the mechanisms and methods of CNSI and ICG are different, but they have high application value in robotic distal gastrectomy, and they are safe and practical. In addition, CNSI was superior to ICG in the number of lymph nodes and micro lymph nodes detected. Overall, CNSI was the best lymph node tracer technique to use during robotic distal gastrectomy in patients with gastric cancer.

## References

[1] Jian-Hui C, Shi-Rong C, Hui W (2016). Prognostic value of three different lymph node staging systems in the survival of patients with gastric cancer following D2 lymphadenectomy. Tumour Biol.

[2] National Health Commission of The People's Republic of China (2019). Chinese guidelines for diagnosis and treatment of gastric cancer 2018 (English version). Chin J Cancer Res.

[3] Mu G, Huang Y, Wei C (2020). Para-aortic lymph node tracing and dissection in advanced gastric cancer: effectiveness of carbon nanoparticles injection through the no.12b lymph node. J Cancer Res Ther.

[4] Patti MG, Herbella FA (2020). Indocyanine green tracer-guided lymph node retrieval during radical dissection in gastric cancer surgery. JAMA Surg.

[5] Wang H, Chen MM, Zhu GS (2016). Lymph node mapping with carbon nanoparticles and the risk factors of lymph node metastasis in gastric cancer. J Huazhong Univ Sci Technol Med Sci.

[6] Landsman ML, Kwant G, Mook GA (1976). Light-absorbing properties, stability, and spectral stabilization of indocyanine green. J Appl Physiol.

[7] Kong SH, Noh YW, Suh YS (2015). Evaluation of the novel near-infrared fluorescence tracers pullulan polymer nanogel and indocyanine green/γ-glutamic acid complex for sentinel lymph node navigation surgery in large animal models. Gastric Cancer.

[8] Chen QY, Xie JW, Zhong Q (2020). Safety and efficacy of indocyanine green tracer-guided lymph node dissection during laparoscopic radical gastrectomy in patients with gastric cancer: a randomized clinical trial. JAMA Surg.

[9] Kwon IG, Son T, Kim H (2019). Fluorescent lymphography-guided lymphadenectomy during robotic radical gastrectomy for gastric cancer. JAMA Surg.

[10] Yan J, Zheng X, Liu Z (2016). A multicenter study of using carbon nanoparticles to show sentinel lymph nodes in early gastric cancer. Surg Endosc.

[11] Lu Y, Wei JY, Yao DS (2017). Application of carbon nanoparticles in laparoscopic sentinel lymph node detection in patients with early-stage cervical cancer. PLoS ONE.

[12] Chaowawanit W, Campbell V, Wilson E (2020). Comparison between laparoscopic and robotic surgery for sentinel lymph node mapping in endometrial cancer using indocyanine green and near infra-red fluorescence imaging. J Obstet Gynaecol.

[13] Roh CK, Choi S, Seo WJ (2020). Indocyanine green fluorescence lymphography during gastrectomy after initial endoscopic submucosal dissection for early gastric cancer. Br J Surg.

[14] Gao H, Liu X, Yu X (2021). Application of nano-carbon tracing technology in thyroid cancer and its relationship with cytotoxic T lymphocyte antigen 4 gene polymorphism. J Nanosci Nanotechnol.

[15] Yu PW, Chen L, Cao H (2016). Expert consensus on robotic surgery for gastric cancer (2015 edition). China Res Hosp.

[16] Ji X, Bu ZD, Li ZY (2017). Prognostic significance of the total number of harvested lymph nodes for lymph node-negative gastric cancer patients. BMC Cancer.

[17] Pan HF, Wang G, Liu J (2017). Robotic versus laparoscopic gastrectomy for locally advanced gastric cancer. Surg Laparosc Endosc Percutan Tech.

[18] Cianchi F, Indennitate G, Trallori G (2016). Robotic vs laparoscopic distal gastrectomy with D2 lymphadenectomy for gastric cancer: a retrospective comparative mono-institutional study. BMC Surg.

[19] Mu G, Huang Y, Wei C (2020). Para-aortic lymph node tracing and dissection in advanced gastric cancer: effectiveness of carbon nanoparticles injection through the no. 12b lymph node. J Cancer Res Ther.

[20] Tian W, Jiang Y, Gao B (2014). Application of nano-carbon in lymph node dissection for thyroid cancer and protection of parathyroid glands. Med Sci Monit.

[21] Kim TH, Kong SH, Park JH (2018). Assessment of the completeness of lymph node dissection using near-infrared imaging with indocyanine green in laparoscopic gastrectomy for gastric cancer. J Gastric Cancer.

[22] Sano T, Coit DG, Kim HH (2017). Proposal of a new stage grouping of gastric cancer for TNM classification: international gastric cancer association staging project. Gastric Cancer.

[23] Kodera Y, Yamamura Y, Torii A (1996). Postoperative staging of gastric carcinoma. A comparison between the UICC stage classification and the 12th edition of the Japanese general rules for gastric cancer study. Scand J Gastroenterol.

[24] Deng J, Zhang R, Pan Y (2014). Comparison of the staging of regional lymph nodes using the sixth and seventh editions of the tumor-node-metastasis (TNM) classification system for the evaluation of overall survival in gastric cancer patients: findings of a case-control analysis involving a single institution in China. Surgery.

[25] Cai J, Ikeguchi M, Tsujitani S (2001). Significant correlation between micrometastasis in the lymph nodes and reduced expression of E-cadherin in early gastric cancer. Gastric Cancer.

[26] Li Z, Ao S, Bu Z (2016). Clinical study of harvesting lymph nodes with carbon nanoparticles in advanced gastric cancer: a prospective randomized trial. World J Surg Oncol.

[27] Huang B, Wang Z, Sun Z (2011). A novel insight of sentinel lymph node concept based on 1–3 positive nodes in patients with pT1-2 gastric cancer. BMC Cancer.

[28] Shoji Y, Kumagai K, Kamiya S (2019). Prospective feasibility study for single-tracer sentinel node mapping by ICG (indocyanine green) fluorescence and OSNA (one-step nucleic acid amplification) assay in laparoscopic gastric cancer surgery. Gastric Cancer.

